# Classification of epileptic EEG signals based on simple random sampling and sequential feature selection

**DOI:** 10.1007/s40708-016-0039-1

**Published:** 2016-02-27

**Authors:** Hadi Ratham Al Ghayab, Yan Li, Shahab Abdulla, Mohammed Diykh, Xiangkui Wan

**Affiliations:** 1 Faculty of Health, Engineering and Sciences, University of Southern Queensland, Toowoomba, QLD 4350 Australia; 2 School of Electrical and Electronic Engineering, Hubei University of Technology, Wuhan, 430068 China

**Keywords:** Electroencephalogram, Epileptic seizures, Simple random sampling, Sequential feature selection, Least square support vector machine

## Abstract

Electroencephalogram (EEG) signals are used broadly in the medical fields. The main applications of EEG signals are the diagnosis and treatment of diseases such as epilepsy, Alzheimer, sleep problems and so on. This paper presents a new method which extracts and selects features from multi-channel EEG signals. This research focuses on three main points. Firstly, simple random sampling (SRS) technique is used to extract features from the time domain of EEG signals. Secondly, the sequential feature selection (SFS) algorithm is applied to select the key features and to reduce the dimensionality of the data. Finally, the selected features are forwarded to a least square support vector machine (LS_SVM) classifier to classify the EEG signals. The LS_SVM classifier classified the features which are extracted and selected from the SRS and the SFS. The experimental results show that the method achieves 99.90, 99.80 and 100 % for classification accuracy, sensitivity and specificity, respectively.

## Introduction

Epilepsy is a disorder which affects the human brain and hugely impairs patients’ daily lives. It is characterized by recurrent and sudden incidence of epileptic seizures [1]. According to an estimation of the World Health Organization, more than 50 million of population are affected by epilepsy [2, 3]. Approximately, almost 1 % population have the neurological disorders [4–6]. It leads to numerous research works to identify epilepsy and related treatments. Electroencephalogram (EEG) signals have been proved as a powerful tool for detecting and diagnosing different neurological diseases. EEG signals are often used to detect and classify epilepsy [7]. It is often difficult for the experts to recognize the people who have a brain disorder through visual inspection of EEG signals [8]. In addition, visual inspection for discriminating EEG signals is a time consuming, error prone, costly process and not sufficient enough for reliable information. The analysis and classification of EEG signals can lead to better diagnostic techniques for brain-related disorders. It is thus important to develop better EEG classification methods.

Many researchers developed new techniques to extract the significant information from EEG signals. The information is used as the input to different classifiers. There are many approaches used to extract the key features as well as to further select features. Most of these fall under five broad categories: time domain, frequency domain, time–frequency domain, traditional non-linear methods and graph theory approaches [9].

One of the methods used in this paper for extracting epileptic EEG data is sample random sampling (SRS) technique. Researchers often applied the SRS in time domain. In this technique, each sample of the population has the same chance to be selected as a subject. The complete process of sampling is done in a single step, with each subject can be selected independently from the other samples of the population [10]. Then, we forwarded all these samples to the sequential feature selection (SFS) method for selecting the best features.

This study uses the selected features as the input for a classifier. One of the most popular classifiers, the least square support vector machines (LS_SVMs) [11], is used to classify EEG data. This technique is used to identify the EEG data from healthy people and epileptic patients for epileptic seizures.

A lot of approaches for EEG signals classification have been developed [12]. There were reported a diverse of classification precisions for epileptic EEG data. Brief discussions of the previous research are provided below.

Gajic et al. [13] extracted different features from time, frequency, time–frequency domain and non-linear analysis.

These features were obtained from sub-bands with good representative characteristics. The researchers reduced the dimension of the features by using scatter matrices. This method yielded 98.7 % accuracy.

An optimum allocation-based principal component analysis method was proposed by Siuly and Li [8] to extract key features for the classification of multi-class EEG signals from epileptic EEG data. They used four different classifiers which were LS_SVM, naive Bayes classifier, *k*-nearest neighbour (KNN) algorithm and linear discriminant analysis, to find out which one was the best classifier. They used four different output coding approaches for the multi-class LS_SVM. These were error correcting output codes, minimum output codes, one versus one (1vs1) and one versus all. That method achieved a 100 % accuracy with LS_SVM_1vs1.

Feature extraction was carried out through an empirical mode decomposition. The extracted features were forwarded to two classifiers, the classification and regression tree and the C4.5 classifiers. The method using the C4.5 classifier suggested by Martis et al. [14] obtained good experimental results of 95.33, 98 and 97 % for accuracy, sensitivity and specificity, respectively.

Chua et al. [15] gained features from raw EEG recordings by using higher order spectra. They used a Gaussian mixture model (GMM) and a SVM classifiers to detect epileptic EEG signals. They achieved average accuracies of 93.11 and 92.56 % for the HOS based GMM classifier and the SVM classifier, respectively, for different EEG classes, such as normal, pre-ictal and epileptic EEGs.

On the other hand, a genetic algorithm (GA) was used by Guo et al. [16] to automatically extract features from EEG data in order to enhance the classifier’s performance, as well as, to reduce the feature’s dimensionality. They used two groups of epileptic datasets. The first group was two classes of healthy people and epileptic patients. The second group was three classes of healthy people, inter-ictal and ictal. The KNN classifier was used in the work to classify the two groups. They gained 88.6 and 99.2 % accuracies for the first group without GA and with GA, respectively. They obtained of a 67.2 % accuracy without GA, and 93.5 % within GA, respectively, for the second group.

Ocak decomposed EEG signals, which were recorded from normal subjects and epileptic patients, by using discrete wavelet transform [17]. An approximate entropy (ApEn) was extracted from the approximation and the detail coefficients. The methodology achieved more than 96 % accuracy.

Srinivasan et al. used the ApEn to extract features and an artificial neural network classifier to identify epileptic EEG signals [18]. That approach achieved a high overall accuracy of 100 %.

Srinivasan et al. also proposed a special type of recurrent neural network, Elman network [19]. They used the feature extracted in time domain and frequency domain as the input to the proposed classifier. The Elman network method yielded a 99.6 % accuracy with a single input feature.

A wavelet transform method was used by Gajic et al. [20] to extract the key features. They also used scatter matrices to reduce the dimensionality of the features. These features were used as the input to a quadratic classifier. The EEG epileptic database was classified into healthy subjects, epileptic subjects during a seizure-free (inter-ictal) and epileptic patients during the seizure activity (ictal). They obtained a 99 % classification accuracy.

Shen et al. [12] proposed a cascade of wavelet-ApEn for feature selection. They used Fisher scores for adaptive feature selection, and SVM for feature classification to detect epileptic seizures. They applied the method to different epileptic EEG recordings: open source EEG data and clinical EEG data. The method obtained the overall classification accuracies of 99.97 and 98.73 %, respectively.

A sampling technique (ST) based on a LS_SVM was proposed by Siuly et al. [21]. Firstly, they used the ST to extract features from two classes of, normal persons with eyes open and epileptic patients during a seizure activity. They applied the LS_SVM to the extracted features. The total classification accuracy by that approach for both the training and testing datasets was 80.31 and 80.05 %, respectively.

Husain and Rao [22] presented an artificial neural network model using back propagation algorithm for the classification of epileptic EEG signals. They decomposed the EEG signals into a finite set of band limited signals termed as intrinsic mode functions. They also applied Hilbert transform on these intrinsic mode functions to calculate instantaneous frequencies. They achieved a 99.80 % overall classification accuracy.

Rückstieß et al. [23] performed a SFS method to select the most representative features at each time step. Each successive features depended on the previous features. All the features were put into one vector and were forwarded to a classifier. This approach was applied for handwritten digits classification and a medical diabetes prediction task.

A sequential floating forward selection (SFFS) algorithm was proposed to detect epileptic seizures in EEG signals by Choi et al. [24]. They selected the most energy power as the features from frequency bands by using the SFFS algorithm. The total accuracy obtained by that method was 97.2 %.

In this study, we developed a new method combining the SRS with the SFS to acquire the best features set, and then we use the features as the input of the LS_SVM classifier for the EEG classification. All the techniques are discussed in Sects. 3 and 4. The conclusion is presented in Sect. 5.

## Experimental data

The data used in this study are open source EEG recordings and are publicly available^1^ [25]. The database includes five sets of EEG recordings (sets **A**–**E**), with each containing 100 single-channel EEG signals of 23.6 s from five separate classes. References [13, 26] presented all details of these datasets from set **A** to **E**. This study selected set **A** which was taken from surface EEG recordings of five healthy people with eye open, and set **E** which was taken from EEG records of five pre-surgical epileptic patients during epileptic seizure activity.

## Methodology

**Fig. 1:**
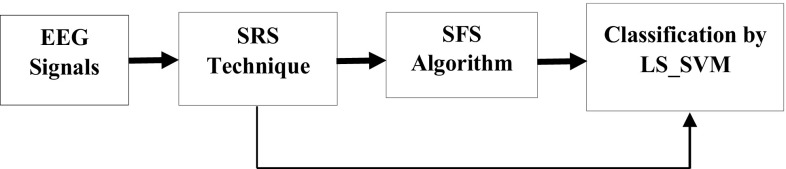
The structure of the proposed system

The big EEG datasets cause the curse of dimensionality and make it difficult to estimate the accuracy of classification from a limited number of samples. This study develops a new structure for classifying epileptic EEG signals, as presented in Fig. 1. This work investigates and explores whether the SRS combined with SFS give the best features for epileptic EEG signals classification.

### Simple random sampling (SRS) technique

SRS technique is a popular type of random or prospect sampling [21]. In this technique, each sample of the population has the same chance of being selected as a subject. We put the number of population in a sample size calculator of the “Creative Research System” (available in sample size calculator online), to determine the sample size for both samples and subsamples. In this work, the dataset used are set **A** and set **E** (repeated). Each set has 100 data files, and each file has 4097 observations.

**Fig. 2:**
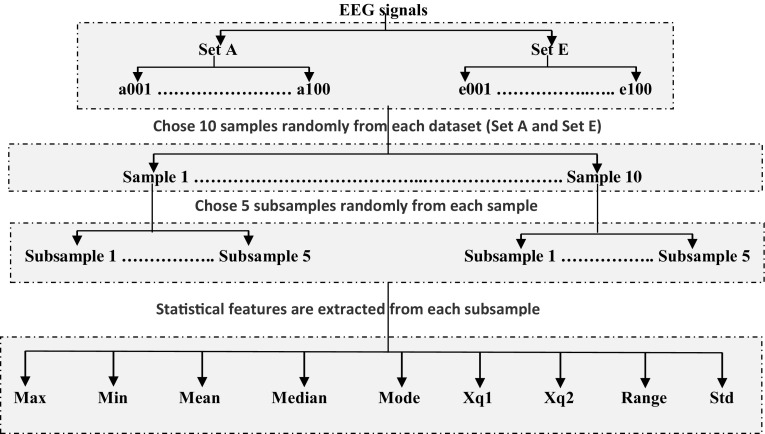
The SRS technique to select samples, subsamples and statistical features

This research uses the sample size calculator to find the sample size needed as well as to find the subsample size. The sizes of the samples and the subsamples in this work are 3288 and 2746, respectively. The sizes were selected because they reflect the limitation of time to select samples and subsamples. Firstly, we randomly select 10 samples from size 3288 for each dataset (set **A** or **E**). Secondly, 5 subsamples are also random chosen from each 10 random samples, with a size of 2746. In each step, this study takes into account a 99–100 % confidence interval and a 99 % confidence level. In the last step, nine statistical features are extracted from each subsample. These features are {maximum value (Max), minimum value (Min), mean value, median value, mode, first quartile (Xq1), second quartile (Xq2), range value and standard deviation (Std)}. Figure 2 shows how samples, subsamples and features are taken from each class. We used MATLAB software package version 8.4, R2014b, for the experiments.

### Sequential feature selection (SFS) algorithms

**Fig. 3:**
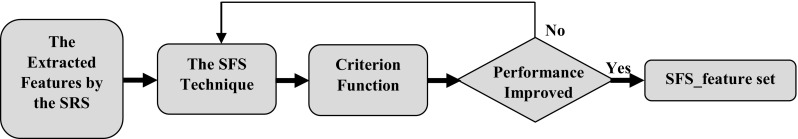
Features selection from the extracted features by the SRS

The SFS is used to reduce the dimensionality of the dataset selected randomly from the SRS. This method is used to generate fewer numbers of uncorrelated variables which are utilized as the features for the better classification of EEG signals. The aim of the presented sequential selection algorithm is to decrease the feature space, **D** = *x*_1_, *x*_2_,…,*x*_*n*_, to a subset of features, **D***− n.* It aims at enhancing or optimizing the computational execution of the classifier, as well as avoiding the curse of dimensionality [27]. This method is used to select a sufficiently reduced subset from the feature space **D** without affecting the performance of the classifier. In order to choose a suitable feature subset size *k*, namely, a criterion function typically estimates the recognition rate of the classifier [28]. The SFS algorithm starts with an empty set ***S***, and progressively fills the set ***S*** through adding features selected by the criterion function [29, 30]. It is searching on the feature space from bottom to up. Figure 3 illustrates how the SFS picks features from the original data. The SFS is applied to select the best features from the statistical features. The criterion is empirically chosen based on the experimental results. In this study, several experiments are made to define the best criterion. The criterion value is calculated based on the statistical relations among the features. Firstly, the Max value is chosen as the criterion as shown in Eq. (1).

1 $$\delta = \rho \sum\limits_{i = 1}^{n} {fs2(i)\quad i = 1,\;2, \ldots ,n},$$

where *δ* refers to the criterion, *ρ* is one of the nine statistical features, *n* is the number of the features and fs2 is the statistical feature set. Secondly, all the features are selected in the same way for Min, Mean, Mode and Std, in order to find the best features by the SFS algorithm. The best features (denoted as SFS_feature) are selected based on Eqs. (2) and (3) as below:

2 $$\delta \le fs2,$$

3 $$\delta > fs2.$$

### The feature set

After decreasing the dimensions of the features through the SFS, the new feature set is forwarded to the LS_SVM classifier. In this study, we obtain a feature set that has 2000 data points of 35 dimensions. These features are divided into two groups, which are the training set and the testing set. The training set is directed to train a classifier. The testing set is employed to evaluate the performance of the methodology and it is utilized as the input of the classifier.

### Least square support vector machines

In this subsection, we briefly review some basic work on LS_SVMs for classification. LS_SVMs are proposed by Suykens and Vandewalle. LS_SVMs are the least square versions of SVMs, which are a set of related supervised learning methods that analyse data and recognize patterns. Moreover, they are used for classification and regression analysis [31]. In this research, the LS_SVM classifier with a radial basis function kernel is used for the classification of epileptic EEG signals. These classifiers can avoid the problem of convex quadratic programming from the classical SVMs by using a set of linear equations [8]. In this paper, the classification is performed by LS_SVMlab (version 1.8) toolbox in MATLAB^2^ [32].

### Performance measures

This subsection presents assessing how the proposed method performs. The assessments include accuracy (also known as recognition rate), sensitivity (or recall) and specificity. The accuracy of a classifier is the percentage of the test set which is correctly classified by the classifier. The sensitivity is referred to the true positive rate which is the proportion of the positive set correctly identified.

The specificity is the true negative average which is the proportion of the negative set correctly identified. The following Eqs. (4)–(6) provide the definitions for the terms [33]:

4 $${\text{Accuracy}} = \frac{{{\text{TP}} + {\text{TN}}}}{{{\text{P}} + {\text{N}}}},$$

5 $${\text{Sensitivity}} = \frac{\text{TP}}{\text{P}},$$

6 $${\text{Specificity}} = \frac{\text{TN}}{\text{N}},$$

where TP is the number of true positives, TN is the number of true negatives and P and N are the positive and negative samples, respectively.

## Results and discussions

In this study, we involved two datasets: sets **A** and **E** as mentioned in Sect. 2. SRS technique was used to extract features from the datasets. This technique selected features randomly by choosing 10 samples from each dataset (sets **A** and **E**). A five subsamples were selected from each sample. From each subsample, nine statistical features, such as minimum, maximum, mean, median, mode, first quartile, third quartile, inter-quartile range and Std were extracted as aforementioned in Sect. 3.1.

A set of features obtained from the SRS included 2000 × 45 dimensions. These features were used in two different ways. Firstly, the statistical features were directly fed to the LS_SVM classifier and yielded the results, as shown in Table 1. Secondly, the SFS based on the criterion was employed to select the key features from the extracted features as mentioned in Sect. 3.2. As shown in the results, the good results of the best features are presented in Table 2. In Table 2, the good results are obtained by using the SRS algorithm and the SFS technique with the LS_SVM classifier depending on the best criterion chosen. Furthermore, the LS_SVM has two important parameters, which are *γ* and *σ*^2^ which should be suitably selected for achieving a desirable performance too. The LS_SVM was affected by the value of these two parameters. This study trained the LS_SVM with different groups of the parameters *γ* and *σ*^2^ to obtain best results. In this proposed method, we conducted with one group of the five EEG datasets and gained the best classification result with sets **A** and **E** when *γ* = 10 and *σ*^2^ = 1 for the two methods applied in this paper. The results of the proposed method were compared with the results that were obtained from the SRS method and the LS_SVM classifier. The experimental results showed that our approach yielded 99.90 % classification accuracy for the epileptic EEG data. Table 3 gives a better view for the results by the two different classification methods. On the other hand, in this study, the evaluation of time complexity between the presented approach and the SRS was conducted.

**Table 1:** Classification accuracy for epileptic EEG signals (sets **A** and **E**)

Statistical parameters	Results (%)
Accuracy	100
Sensitivity	100
Specificity	100

**Table 2:** Experimental results using different statistic features as the criterion

Choose criterion	Accuracy (%)	Sensitivity (%)	Specificity (%)
Mean ≥ fs2 (SFS_feature)	99.90	99.80	100.00
Mean ≤ fs2 (SFS_feature)	98.90	98.00	99.80
Max ≤ fs2 (SFS_feature)	97.20	100.00	94.40
Min ≥ fs2 (SFS_feature)	99.10	99.20	99.00
Mode ≥ fs2 (SFS_feature)	97.70	95.40	100.00
Median ≤ fs2 (SFS_feature)	95.30	92.80	97.80
Std ≥ fs2 (SFS_feature)	95.60	91.20	100.00

**Table 3:** Comparison of the results and time complexity for the proposed method with other methods

Methods	Accuracy (%)	Sensitivity (%)	Specificity (%)	Time (s)
SRS_LS_SVM	100.00	100.00	100.00	1.52
The proposed method with the best criterion (SRS_SFS_LS_SVM)	99.90	99.80	100.00	0.16

The SRS_SFS_LS_SVM method took 0.16 s to classify the extracted features in Sect. 3.2. While the SRS_LS_SVM tackled the same features with 1.52 s as shown in Table 3. The performance of the proposed method is also compared with two existing methods in the literature. For fair comparison, the same dataset was used in comparison. The results show that the proposed method outperforms over the other two existing methods: a Huang–Hilbert transform and an artificial neural network model by Husain and Rao [22] and a ST and LS_SVM methods by Siuly et al. [21]. The performance comparison of the proposed method with the two reported methods to classify sets **A** and **E** is shown in Table 4. Husain and Rao in 2014 applied a Huang–Hilbert transform and an artificial neural network model on sets **A** and **E** (the same datasets used in this paper). They achieved a 99.80 % classification accuracy. While Siuly et al. in 2009 obtained 80.05 % classification accuracy when they used a ST and the LS_SVM methods to classify the EEG signals for the same datasets. Moreover, the proposed method gains a 99.90 % classification accuracy for the same group of datasets. The results shown that the proposed technique in this paper has the potential to classify the EEG signals from healthy people and epileptic patients using the extracted and selected features from the SRS and SFS techniques.

**Table 4:** Comparison of performance of our proposed method with two recently reported methods for sets **A** and **E** of the EEG epileptic database

Different methods	Accuracy (%)	Sensitivity (%)	Specificity (%)
The proposed method with the best criterion (SRS_SFS_LS_SVM)	99.90	99.80	100
A Huang–Hilbert transform and an artificial neural network model [22]	99.80	99.75	100
A sampling technique and LS_SVM method [21]	80.05	74.97	87.70

## Conclusions

This research concentrates on two classes of EEG signals from healthy people and epileptic patients. The study presents a SRS_SFS method to extract and select the key features for classifying EEG signals into two classes. The LS_SVM classifier is used to classify two-category EEG data after the feature extraction and selection. This method yields the results of 99.90, 99.80 and 100 % for classification accuracy, sensitivity and specificity, respectively. In addition, the proposed method is faster than the SRS technique. It means that the SRS_SFS is useful for extracting and selecting the EEG features. To sum up, the proposed method is very efficient for analysing and classifying epileptic EEG signals. It will be also useful for the classification of other biomedical data.

## Footnotes

1 http://www.meb.unibonn.de/epileptologie/science/physik/eegdata.html.

2 http://www.esat.kuleuven.ac.be/sista/lssvmlab/.
